# Comparison of the quality of life of patients with liver cirrhosis before and during the COVID-19 lockdown in Slovakia

**DOI:** 10.1038/s41598-023-29510-2

**Published:** 2023-02-11

**Authors:** Ľ. Skladaný, D. Líška, E. Liptáková, T. Tapajčiková, J. Vnenčaková, T. Koller

**Affiliations:** 1 HEGITO (Div Hepatology, Gastroenterology and Liver Transplant), 2nd Department of Internal Medicine, Faculty of Medicine, Slovak Medical University, F. D. Roosevelt Teaching Hospital, Banská Bystrica, Slovakia; 2 2nd Department of Internal Medicine, Faculty of Medicine, P. J. Safarik University, Kosice, Slovakia; 3 Department of Physical Education and Sports, Faculty of Arts, Matej Bel University, Tajovského 40, 974 01 Banská Bystrica, Slovakia; 4 Department of Applied Mathematics and Business Informatics, Faculty of Economics, Technical University of Košice, Košice, Slovakia; 5 Faculty of Healthcare, Slovak Medical University in Bratislava, Banská Bystrica, Slovakia; 6 Gastroenterology and Hepatology Subdiv, 5th Department of Internal Medicine, University Hospital Bratislava, Comenius University Faculty of Medicine, Bratislava, Slovakia

**Keywords:** Cancer, Medical research

## Abstract

Liver cirrhosis is associated with a poor quality of life (QOL). The COVID-19 pandemic has led to several restriction measures and psychosocial consequences whose impact on QOL has combined with that of cirrhosis in an unknown way. Therefore, we have used our cirrhosis registry to assess the quality of life before the pandemic (on the first admission to the tertiary liver unit) and during the most pronounced phase of the first lockdown. In this cross-sectional study conducted during the first lockdown in Slovakia (from April to May 2020), we have repeated the QOL measurement of QOL in cirrhotic patients previously enrolled in the RH7 registry. Patients who were alive (according to the national registry of deaths) were identified and contacted by phone with a structured and standardized interview led by trained professionals. The tool used for both QOL measurements (at enrolment in RH7 and during lockdown) was a standardized and validated EuroQOL-5D (EQ-5D) questionnaire. The study included 97 patients, of which 37 (38.1%) were women and 60 (61.9%) were men. Responses were achieved from 75 patients (68.18%). In general, patients scored their quality of life significantly higher during the pandemic compared to examination at admission to RH7 (that is, at admission to our tertiary liver unit with cirrhosis) (*p* = 0.005). In particular, of the domains included in EQ-5D: (1) self-care was better during lockdown compared to the first record on admission to RH7 (*p* < 0.001). (2) the ability to perform daily activities has also improved during lockdown (*p* = 0.002). On the other hand, (3) pain and discomfort did not change significantly during the lockdown compared to the previous measurement (*p* = 0.882). (4) anxiety and depression were lower during lockdown compared to admission to RH7 (*p* = 0.01). The quality of life in patients with liver cirrhosis was better during the lockdown of SARS-CoV-2 compared to the previous measurement at admission to the tertiary liver unit.

## Introduction

Liver cirrhosis is the final stage of chronic liver diseases of various aetiologies. It is characterized by loss of functional parenchyma, compensatory regeneration, and fibrosis, leading to disorganization of liver architecture and function^1,2^. The global prevalence of cirrhosis has ranged from 0.15 to 0.27%, and in Slovakia, it is the highest in the world^1,3–6^. The most common causes of cirrhosis are alcohol-associated liver disease (ALD), non-alcoholic or metabolic-associated fatty liver disease (NAFLD, MAFLD), autoimmune syndromes (autoimmune hepatitis, primary biliary cholangitis, primary sclerosing cholangitis, etc.), hepatitis B and hepatitis C, and many others^7–9^.

Cirrhosis patients have a poor prognosis that is primarily mediated by acute decompensation, especially its subtype—acute on chronic liver failure (ACLF)^10–12^. Liver cirrhosis is associated with human suffering that is not captured by disease-directed diagnostic tools, but rather in the domain of patient-reported outcomes (PRO), the most frequent PRO is the quality of life (QOL)^13–16^. In cirrhosis, QOL is the metric of well-being associated with the severity of the disease^17–19^. The World Health Organization has defined QOL as a subjective assessment of perception of its reality in health, the synonym being satisfaction with life^20–22^. A synonym for the term QOL is the assessment of satisfaction with life^23^. QOL has become an important topic in the healthcare of patients with liver cirrhosis^17^. Health-related quality of life includes factors that are part of an individual’s health^24^.

The pandemic caused by SARS-CoV-2 (COVID-19) severely affected all aspects of our lives and the healthcare system^25–27^. The Slovakian government declared restrictive lockdown regulations to control the spread of the infection in March 2020. Gatherings of more than six people and all mass events were banned. All universities, primary and secondary schools, and fitness and wellness centres had to be closed^28^. 

In our previous study, we evaluated the impact of the lockdown on mortality in patients with cirrhosis, registered in the Cirrhosis Registry (RH7)^29^. Our results pointed to a distorted path to specialized liver care with fewer visits and admissions to a tertiary liver unit. Additionally, since the liver unit centre was separated from our locked patients, we were unable to perceive their personal experiences during the most demanding period, we decided to reach out and take advantage of the previously measured quality of life. Using available telemedicine at hand (telephone survey), we intended to compare the pre-pandemic QOL measured at baseline (when entering RH7) with the repeated assessment during the lockdown. 

The main objective of our study was to evaluate the quality of life in cirrhosis patients from the RH7 Cirrhosis Registry during a lockdown and to compare the results with the quality of life measured previously, at their enrolment in RH7.

## Methods

The study is a combination of the registry study (RH7, NCT04767945) in which QOL is a core baseline parameter, with the cross-sectional investigation determining QOL during the lockdown. The main objective of this study was to compare the quality of life at the time of enrolment in our registry with the quality of life measured later during a lockdown. The study population consisted of patients with liver cirrhosis, registered in the Cirrhosis Registry (RH7)- RH7 has been operating in our liver unit since 2014 and enrols consecutive consenting adults admitted to the hospital with liver cirrhosis^30^. Patients with terminal liver disease or severe comorbidity, both of which can limit short-term life expectancy, are not included in RH7. Among other baseline parameters recorded at admission to our liver unit and in RH7, a generic EuroQol-5D questionnaire (EQ-5D) is administered; this tool was selected for its validity and reproducibility in liver diseases and for its brevity^31^.

At the first admission to our liver unit with liver cirrhosis, patients completed their first EQ-5D and the results were recorded in the RH7 core dataset. Subsequently, in the second part of the study, which was conducted during the first lockdown in Slovakia in April–May 2020, EQ-5D was administered by healthcare professionals who contacted all survivors of RH7 identified in the National Registry of Deceased by an investigator. The interviewing physicians were trained for a structured dialogue whose content and explicit reading were approved by the Ethics Committee of our academic hospital F. D. Roosevelt Teaching Hospital under number 9/2020, Banská Bystrica, Slovakia. The physicians introduced themselves (most of them became familiar with patients during the previous hospitalization), asked permission to continue, and explained the purpose of the call and the study, which was to see how they are and to repeat the formal determination of QOL by EQ-5D (EQ-5D was explained to be the same as previously—at the entrance to RH7). After a pause, the interviewing physicians asked for explicit consent and wrote it down; only then did they proceed to EQ-5D. The physicians then recorded all of the responses from the patients using a pen-and-paper method. If there was no response to the first telephone call, the call was repeated three times in three consecutive days; after that, the result was recorded as ‘No Connect’. If the patient contacted declined participation, the result was recorded as ‘Declined to participate’. The reasons for this were not recorded.

### EuroQOL-5D (EQ-5D)

EuroQOL-5D (EQ-5D) is a generic questionnaire to measure QOL which has been validated in various diseases^32^. EQ-5D has been shown to be valid in liver patients comparable to our cohort^33^. EQ-5D measures five aspects underlying quality of life: mobility, self-care, daily activities, pain, anxiety, and depression. The EQ-5D total score is defined as a score between 5 and 25 recorded by an individual for his current overall quality of life. A pilot test was conducted before the start of the study to determine the feasibility of submitting a questionnaire. The owner of EQ-5D has kindly granted HEGITO permission to use it.

### The sample (Flowchart)

**Figure 1:**
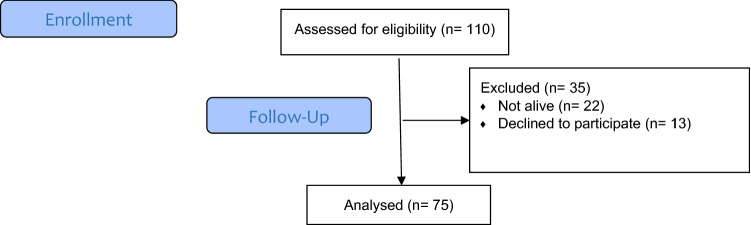
Flow chart.

The study included 97 patients, of which 37 (38.1%) were women and 60 (61.9%) were men. An additional 22 patients (not yet listed in the National Registry of Deceased) were identified as dead during the study (Fig. 1). The characteristics of the sample are shown in Table 1.

**Table 1:** Baseline participants’ characteristics.

Number of participants	97
Gender	
Women	37 (38.1%)
Men	60 (61.9%)
Age (years), mean (SD)	55.9 (± 11.8)
Weight (kg), median (IQR)	76.5 (± 25.3)
Height (cm), mean (SD)	171.6 (± 10.0)
BMI (kg/m^2^), median (IQR)	26.2 (± 7.3)
Etiology of cirrhosis	
ALD, n/%	69 (71.13%)
Other, n/%	28 (28.87%)
MELD, median (IQR)	15.0 (± 9.0)
Child–Pugh Score, median (IQR)	8.0 (± 4.0)
Liver frailty index, mean (SD)	4.48 (± 0.94)

*BMI* body mass index, *ALD* alcohol liver disease, MELD- Model for End-Stage Liver Disease. Variables with skewed data distribution are presented as median and interquartile range (IQR). Age was recorded at the baseline—entry into registry RH7. LFI was calculated at https://liverfrailtyindex.ucsf.edu/

### Statistical analysis

Patient data were recorded to an Excel spreadsheet and subsequently subjected to statistical analysis using the IBM SPSS software package. The difference between the pre-pandemic period and the pandemic period in the EQ-5D individual score and the total score was analysed using the non-parametric paired Wilcoxon Signed Ranks test because of the ordinal type of data or data was not normally distributed.

### Ethical approval and consent to participate

All procedures performed were in accordance with the ethical standards of the institutional and/or national research committee and with the 1964 Helsinki Declaration and its later amendments or comparable ethical standards. Informed consent was obtained from all individual participants involved in the study. The study was approved by the Ethics Committee of the Roosevelt Hospital in Banská Bystrica under no. 9/2022.

## Results

97 patients from the RH7 cirrhosis registry were enrolled, aged 55.9 (± 11.8) years with 38.1% of them women. The median MELD score was 15.0 (IQR 9.0 scores typical for decompensated cirrhosis), and the median Child–Pugh score was 8.0 (IQR 4.0). These scores are characteristic of advanced cirrhosis (Table 1). The median time between the two QOL examinations was 184 days. The baseline QOL was significantly worse than QOL assessed during the lockdown (Table 2). Three of the EQ-5D domains differed significantly between the two time points: self-care improved (*p*  < 0.001), usual activities (*p*  = 0.002), and anxiety/depression improved (*p*  = 0.012). These three domains improved during the lockdown (Table 2). Improvement in mobility observed during lockdown did not reach statistical significance (*p* = 0.103). There was no change in the pain and discomfort (*p* = 0.882). Detailed results are shown in Table 2.

**Table 2:** Relative frequencies of the EQ-5D individual scores for the five domains during the first hospitalization and during the lockdown period—authors’ survey results.

Item of the EQ-5D	Period	Mean score	Z-score*	*p* value*	EQ-5D score				
					(1) (%)	(2) (%)	(3) (%)	(4) (%)	(5) (%)
Mobility	Baseline	2.082	− 1.631	0.103	42.3	29.9	13.4	6.2	8.2
	Lockdown	1.856			48.5	27.8	14.4	8.2	1.0
Self-care	Baseline	1.845	− 3.462	< 0.001	61.9	16.5	6.2	6.2	9.3
	Lockdown	1.320			82.5	7.2	6.2	4.1	0.0
Usual activities	Baseline	2.082	− 3.077	0.002	51.5	17.5	13.4	6.2	11.3
	Lockdown	1.588			66.0	13.4	16.5	4.1	0.0
Pain/ Discomfort	Baseline	1.814	− 0.148	0.882	46.4	33.0	14.4	5.2	1.0
	Lockdown	1.804			55.7	16.5	19.6	8.2	0.0
Anxiety/ Depression	Baseline	1.639	− 2.501	0.012	61.9	18.6	15.5	2.1	2.1
	Lockdown	1.340			77.3	14.4	5.2	3.1	0.0

Baseline period—during the first hospitalization. Lockdown period—during the pandemic. EQ-5D individual scores are on a scale from 1 (no problem) to 5 (maximum problems).

*According to paired Wilcoxon Signed Ranks Test.

**Figure 2:**
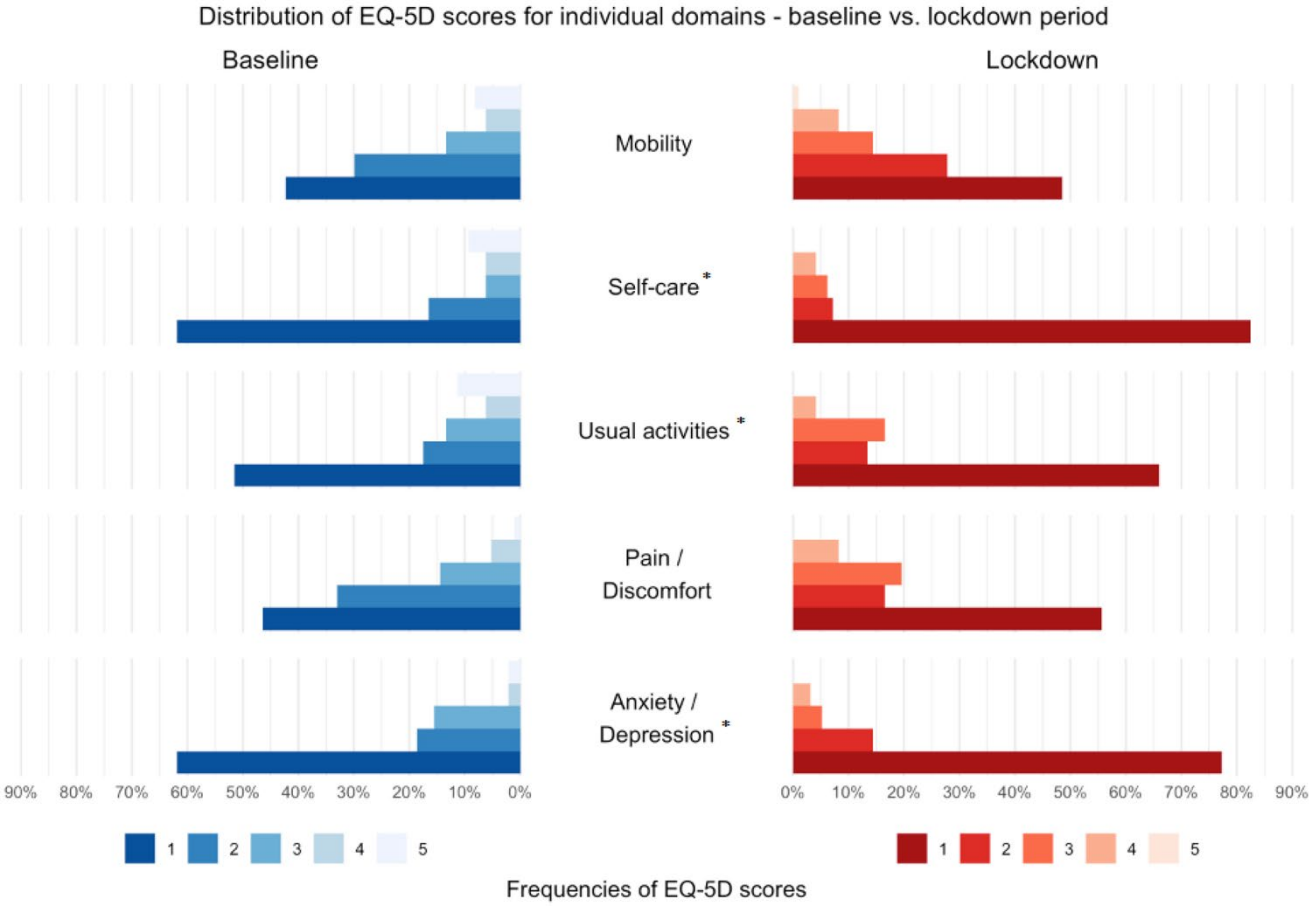
Distribution of the EQ-5D scores for individual domains—baseline versus lockdown period. *Note*: Baseline period—during the first hospitalization. Lockdown period—after discharge from the hospital, during the pandemic. EuroQOL scores are on a scale from 1 (no problem) to 5 (maximum problems).

The distribution of the individual scores for the five domains during the first hospitalization and during the pandemic period are graphically displayed in Fig. 2.

**Figure 3:**
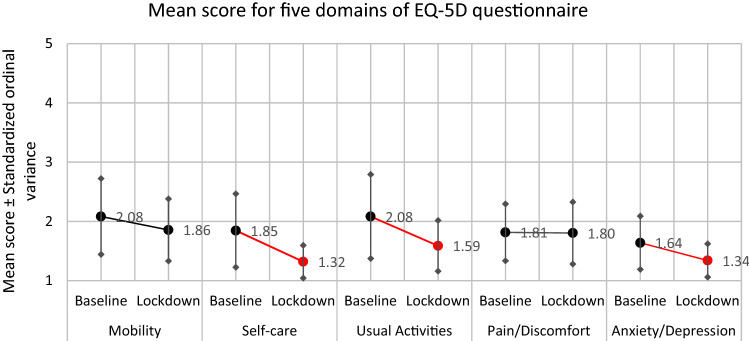
Mean score with the standardized ordinal variance for domains of the EQ-5D questionnaire—baseline versus lockdown period. *Note* Baseline period—during the first hospitalization; Lockdown period—during the pandemic. EuroQOL scores are on the scale from 1 (no problem) to 5 (maximum problems). Red line with red point show statistically significant changes between two time periods—improvement in lockdown period.

On the visual analogue scale for overall QOL feeling, there was a significant increase in the score in favour of the lockdown period meaning that QOL increased (*p* = 0.017). The EuroQOL Final Score was a significant improvement during the pandemic compared to the baseline, and it was consistent in both female and male patients (*p* < 0.005, Table 3, Fig. 3).

**Table 3:** EQ-5D total score during the first hospitalization and during the lockdown period according to the gender of patients.

Patients	Period	Mean total score	Mean difference	Z-score*	*p* value*
Women	Baseline	10.41	− 2.703	− 3.052	0.0023
	Lockdown	7.70			
Men	Baseline	8.82	− 0.783	− 1.019	0.3083
	Lockdown	8.03			
All	Baseline	9.42	− 1.515	− 2.757	0.0058
	Lockdown	7.91			

Overall, and in women, the total QOL score improved during the lock-down as compared to the first hospital admission.

Baseline period—during the first hospitalization. Lockdown period—during the pandemic.

*According to paired Wilcoxon Signed Ranks Test.

**Figure 4:**
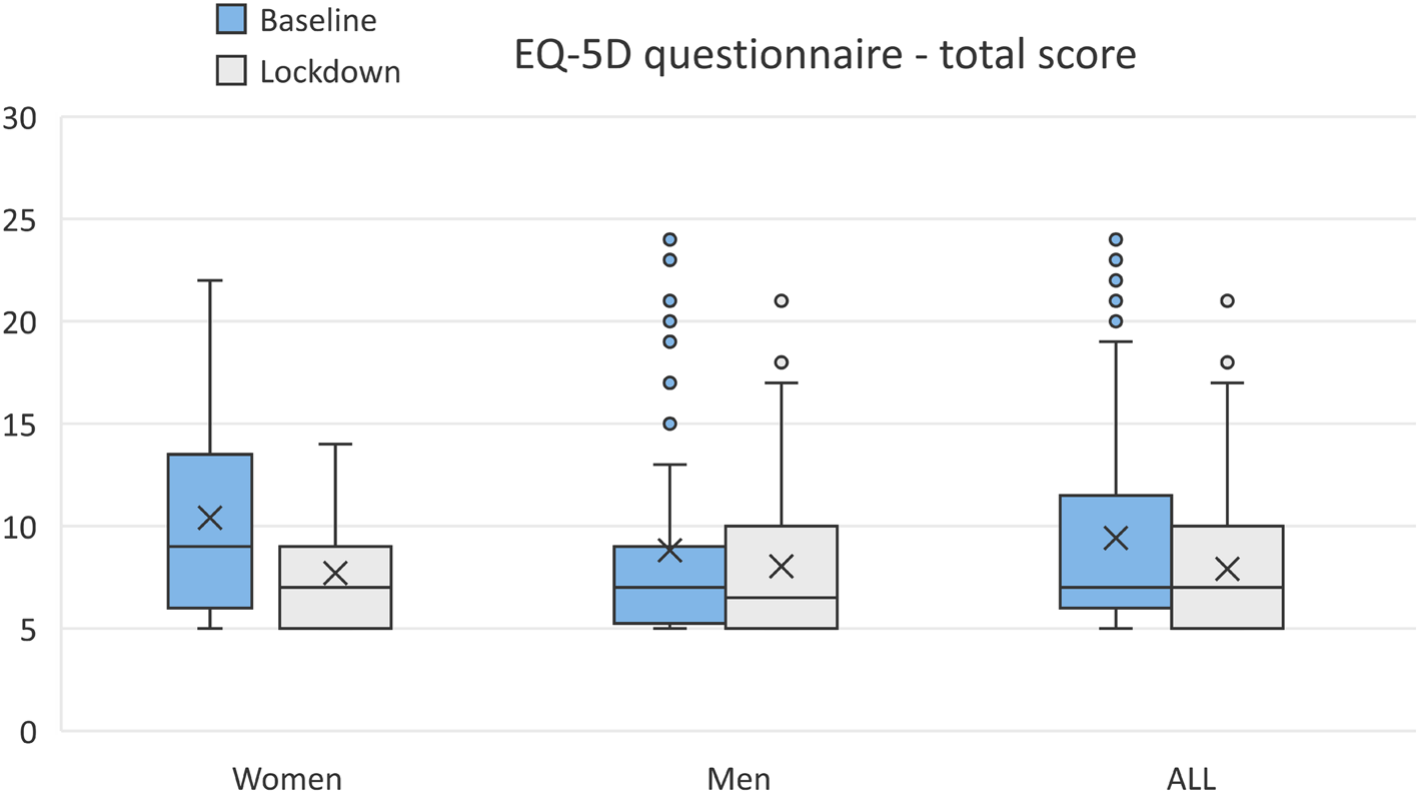
EQ-5D total score distribution by gender—baseline and lockdown period (Women, *p* = 0.0023; Men, *p* = 0.2498; All, *p* = 0.00501 Note: Baseline period—during the first hospitalization. Lockdown period—during the pandemic. The lower total score signifies a better quality of life. A total score of 5 means the best quality of life, total score 25 means the worst quality of life.

Figure 3 shows the mean score for each domain of the EQ-5D questionnaire along with the standardized ordinal variance for the two time periods, the baseline and the lockdown. The distribution of the total scores of the EQ-5D questionnaire during baseline and lockdown period by gender is graphically displayed in the Fig. 4.

## Discussion

The teleological background of this study dwells in our use of the previously measured QOL in the RH7 Cirrhosis Registry RH7 and comparing it with repeated QOL measurement during the once-in-a-lifetime extreme social context, the unprecedented psychosocial environment inflicted by the lockdown. Data were recorded on QOL evaluations in all our patients admitted to the hospital with liver cirrhosis ever since 2014. For the sake of brevity for registry purposes, construct validity in cirrhosis and Europeans, reproducibility, and comparability throughout diseases, and generic EuroQOL were obtained to determine QOL in patients with liver cirrhosis. EuroQOL has higher sensitivity and specificity than cirrhosis-specific instruments. It was developed by Younoussi^34^ and reported to have a cumulative advantage of reproducibility, validity, validity in Europeans, validity in liver diseases, comparability with other diseases, and brevity^32,35–37^. 

After the initial shock from the pandemic, we were cut off from most of our outpatients, including patients with cirrhosis who were seen regularly after discharge. Our RH7 registry included hospitalized patients mainly with decompensated cirrhosis and a poor prognosis. At first, we felt obliged to actively contact them during lockdown to learn how they were and help them in case of need. At this stage, we decided to expand our effort to a formal study using EuroQOL. In general, cirrhosis is associated with a low quality of life^16^. It is chronic and progressive, leading to a deterioration in mental and physical functioning and significantly impacting the quality of life. Additionally, a disease-driven decrease in quality of life can be further multiplied by other individual psychological and psychosocial factors. It is increasingly recognized as an important outcome of cirrhosis. The use of a quality-of-life questionnaire can identify high-risk patients with poor quality of life and a worse prognosis. Understanding the importance of quality of life is an important factor in providing high-quality management^15^. 

Our main finding of an improvement in overall quality of life during lockdown was a great surprise. There are several generic explanations for these findings. First, at baseline, we meet our patients in very poor physical condition to ensure the quality of life at its nadir^38^. Patients with decompensated cirrhosis are usually referred to our tertiary liver unit with a liver transplant program when first and second-line therapeutic options have failed. This may be not only unabated physical suffering from decompensating events (encephalopathy, ascites, infections, frailty, bleeding, etc.) but also mental suffering. Several factors that affect the quality of life have been identified in patients with liver cirrhosis. In a study by Parkash et al.^39^ low quality of life was associated with a low level of haemoglobin, serum albumin, and previous decompensation of liver cirrhosis. In a study by Solà et al.^40^ hyponatremia and oedema were the main risk factors associated with a poorer quality of life. A study by Les et al.^41^ identified ascites, hypoalbuminemia, minimal liver encephalopathy, and anaemia as potential factors of poor quality of life.

In our study, some factors may have led to an improvement in quality of life during the lockdown. First, the impact of treatment and recovery from the initial complication. Furthermore, each patient was educated about the need for optimal nutrition and the benefits of exercise that could lead to a better quality of life. A positive change in the functional capacity of the muscles could also affect daily activities. Second, a temporary increase in anxiety and depression during the first hospitalization^42^ was probably followed by a gradual decrease related to recovery from acute complications. Furthermore, in some patients, inner reconciliation with the diagnosis could have led to acceptance, regained hope, and self-empowerment. Thus, both the above-mentioned physical and psychological mechanisms would understandably improve after discharge (33). Furthermore, several other unmeasured factors could have contributed to our findings: greater care and interest from family members during the pandemic and a significant decrease in daily stressors, such as going to work or having the responsibility for administrative tasks.

The impact of social isolation on quality of life is enormous and significant^43,44^. On the other hand, patients with liver cirrhosis may have had some degree of social isolation before the pandemic. An increased incidence of anxiety and depression during the first hospitalization can be related to anxiety about the disease^42^, and a gradual decrease may be related to the alleviation of the disease. Stress during the first hospitalization and fear of an unknown illness can significantly affect the overall quality of life during the first hospitalization and can be an explanation compared to the quality of life during a pandemic. The diagnosis of a serious disease such as liver cirrhosis can cause a negative reaction in patients, which will significantly affect the quality of life of patients; however, reconciliation with the diagnosis can lead to greater resistance in some patients. At admission to our liver unit, patients might be at the peak of their psychological suffering as well—such as fear of death, uncertainty if there are any further therapeutic options left, and would she or he be a suitable candidate for third-line therapeutic options such as liver transplantation. Both of the above-mentioned (physical and psychological) causes of decreased quality of life would be understandably improved after discharge from our unit by an improvement in physical state, by psychological counselling, as well as by a regained hope (33). Better care and interest from family members during the pandemic could also help improve the quality of life. The disappearance of some stressors, such as going to work during a pandemic, may have contributed to a paradoxical improvement in patient quality. Finally, there is yet another possible explanation for the improved QOL during lockdown: a shift of the psychological focus from a purely personal domain (my disease, my pain, my uncertainty, my mortal being, etc.) to the higher-order domains such as the suffering of others, the fate of the whole society or the world, future of the globe, and how can I be of help to others^45,46^. These higher-order preoccupations of patients could have overshadowed their suffering to the extent that we have been able to detect an improvement in QOL. Domains significantly improved during lockdown compared to a hospital stay (self-care, usual activities, and anxiety/depression) could be similar to concepts previously described in the classical literature in Aldous Huxley’s Psychological causes of war and Paolo Coelho’s Veronika Decides to Die^47^. They have contemplated the reasons for the (real or fictional) reduction in suicide rates during wartime and cured psychiatric illnesses *against the* immediate threat of death. At this point, with some degree of an overstatement, we can safely hypothesize that the quality of life of our patients improved as a direct psychological consequence of the pandemic.

Another factor associated with a lower quality of life in patients with liver cirrhosis is malnutrition^48^. Malnutrition is associated with an increased risk of mortality, hospitalizations, portal hypertension, and infectious complications^49,50^. In our study, patients were informed about the need for a healthy lifestyle that could affect their quality of life during a pandemic. Sarcopenia is a significant factor that adversely affects the quality of life in patients with liver cirrhosis^51–53^. During the pandemic, overall physical activity decreased, which may have led to a higher prevalence of sarcopenia. However, compared to the first hospitalization, physical activity was higher, likely contributing to improved quality of life in cirrhosis. Sarcopenia is also closely related to the appearance of frailty. Nishikawa et al.^54^ studied the relationship between frailty and quality of life in patients with cirrhosis. All aspects of SF-36 were associated with frailty syndrome (*p* < 0.0001). A change in the functional capacity of the muscles had a negative effect on the daily activities of the patients. Da Silva Vieira et al.^55^ tested functional capacity in patients with cirrhosis and found a correlation between the quality of life and the result of a 6 min walk test.

How can we test the hypotheses generated by our results? First, we can systematically start repeating measurements of the QOL at discharge and further on to see trends. Second, we can ask our patients for reasons for their perceived improvements in the QOL during the lockdown while being aware of the inherent limitations that both of these attitudes have.

Our study was associated with several limitations. Twenty-two patients died during the study and the inability to evaluate the data may have affected the results. The study was monocentric with a focus on the central part of Slovakia. The study was carried out in Slovak patients; To objectively assess the quality of life with liver cirrhosis during a pandemic, it would be necessary to include more patients from different centres and countries.

## Conclusion

The quality of life measured by the EuroQOL questionnaire in patients hospitalized in the tertiary liver unit with decompensated cirrhosis was low. After discharge and during the toughest pandemic-associated lockdown, the QOL has improved. The explanation is not straightforward, and further investigation of the causes of this unexpected finding is warranted.

## Data Availability

The datasets used and/or analysed during the current study are available from the corresponding author upon reasonable request.
